# Transcatheter Electrosurgery for Coronary Access After TAVI-in-TAVI

**DOI:** 10.1016/j.jaccas.2025.106733

**Published:** 2026-01-19

**Authors:** Dyhia Zourane, Benjamin Benharoun, Jérémie Abtan, Sandra Zendjebil, Jules Mesnier, Fouad Saadi, Phalla Ou, Laurent Feldman, Marina Urena, Nathan El Bèze

**Affiliations:** a Department of Cardiology, Bichat Claude Bernard Hospital–Paris City University, Paris, France; b Department of Radiology, Bichat Claude Bernard Hospital–Paris City University, Paris, France

**Keywords:** aortic stenosis, coronary access, electrosurgery, percutaneous coronary intervention, structural heart intervention, valve-in-valve

## Abstract

**Background:**

Transcatheter aortic valve implantation (TAVI) is increasingly performed, with TAVI-in-TAVI reinterventions becoming more frequent. Coronary access after TAVI-in-TAVI can be severely limited by neoskirt formation.

**Case Summary:**

A 74-year-old man with extensive comorbidities and prior TAVI-in-TAVI (Evolut and Sapien valves) presented with chest pain and troponin elevation. Angiography showed severe left main artery and left anterior descending artery stenoses, but selective coronary engagement was impossible given complete neoskirt obstruction. Using transcatheter electrosurgery, a Steelcore guidewire insulated in a microcatheter was energized to perforate the neoskirt. Percutaneous coronary intervention with drug-eluting stents was successfully completed, with an excellent final result.

**Discussion:**

To our knowledge, this is the first report of electrosurgical neoskirt perforation to enable coronary revascularization after TAVI-in-TAVI.

**Take-Home Messages:**

Electrosurgery can safely perforate a neoskirt, restoring coronary access. This novel strategy may become essential as TAVI-in-TAVI procedures increase.

## Visual Summary

**Figure:**
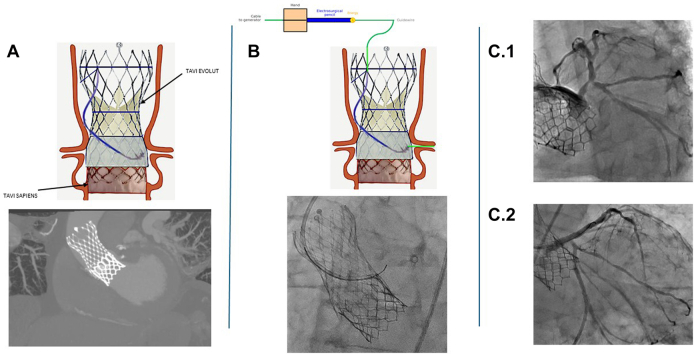

Transcatheter aortic valve implantation (TAVI) has become an effective alternative to surgical valve replacement for patients with severe aortic stenosis. The growing number of TAVI procedures in recent years is explained by expanded indications, now including intermediate- and low-risk surgical patients, as well as ongoing technological advancements in percutaneous valve interventions. With the increase in TAVI procedures, reinterventions are becoming more frequent, often necessitating TAVI-in-TAVI.

**Take-Home Messages**

• Double TAVI can create a neoskirt that completely impedes coronary access, posing major challenges for revascularization despite adequate coronary perfusion.
• Transcatheter electrosurgery provides a safe and effective solution to re-establish coronary access when standard percutaneous techniques fail.

Coronary access after TAVI is challenging. The main predictors of difficult coronary access are anatomical, including high valve implantation, a high ratio between valve size and the sinuses of Valsalva, and the use of a self-expanding valve.^1^

**Visual Summary:**
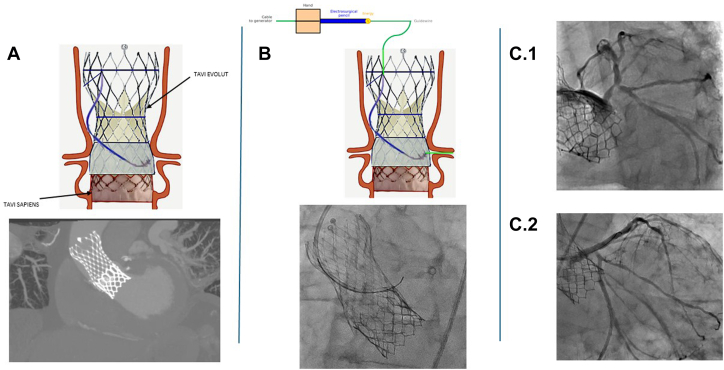
Electrosurgical Neoskirt Perforation to Enable Coronary Revascularization After TAVI-in-TAVI (A) Failed coronary cannulation due to neoskirt obstruction after TAVI-in-TAVI. (B) Monopolar electrosurgery applied to a guidewire enables controlled perforation of the neoskirt after TAVI-in-TAVI, restoring coronary access. (C.1) Baseline coronary angiography, nonselective injection. (C.2) Final angiography shows successful coronary revascularization after neoskirt perforation and PCI. PCI = percutaneous coronary intervention; TAVI = transcatheter aortic valve implantation.

Moreover, de novo coronary lesions are not uncommon after TAVI. Post-TAVI acute coronary syndromes occur with an incidence of 4.7% at 2 years, with a strong predominance of non–ST-segment elevation myocardial infarction (89.6% of cases). Risk factors such as pre-existing coronary artery disease, diabetes, or chronic renal failure justify close monitoring and follow-up of these patients.^2^

To address this challenge, innovative strategies such as transcatheter electrosurgery play a crucial role in optimizing clinical outcomes. Transcatheter electrosurgery employs high-frequency alternating current to vaporize, cut, or traverse tissues remotely without surgical exposure.^3^^,^^4^ A well-known use of transcatheter electrosurgery is the BASILICA (bioprosthetic or native aortic scallop intentional laceration to prevent iatrogenic coronary artery obstruction) technique, which consists of lacerating a valve leaflet in front of a threatened coronary artery.

Here, we present a complex case of percutaneous coronary revascularization in a patient with double TAVI implantation, highlighting the utility of transcatheter electrosurgery in overcoming anatomical and procedural barriers.

## History of Presentation

A 74-year-old man was referred to the emergency department owing to chest pain and tightness that had persisted for 4 days before presentation. The patient had a significant medical history, including right single lung transplantation for fibrosing interstitial lung disease, end-stage chronic kidney disease, a right superficial middle cerebral artery stroke secondary to carotid atherosclerosis, prostate adenocarcinoma treated with radiotherapy, and a frontal falcine meningioma.

Regarding cardiovascular history and risk factors, he had coronary artery disease with prior stenting of the mid left anterior descending artery (LAD), a TAVI using a 29-mm Evolut valve with supra-annular implantation, which required a 26-mm Edwards Sapien TAVI-in-TAVI in the same procedure in 2022. He also had diabetes mellitus, hypertension, hyperlipidemia, and was a current smoker.

## Investigations

On arrival, the patient was pain free and stable, with a blood pressure of 112/78 mm Hg, heart rate of 86 beats/min, and oxygen saturation of 98% on room air. Physical examination was normal except for basal crackles.

The admission electrocardiogram showed sinus rhythm with a newly detected left bundle branch block. High-sensitivity troponin peaked at 1,900 ng/L (reference range: <14 ng/L). Transthoracic echocardiography demonstrated a preserved left ventricular ejection fraction of 50% with intraventricular dyssynchrony, no regional wall motion abnormalities, a functioning TAVI, and normal right ventricular function.

## Management

**Figure 1:**
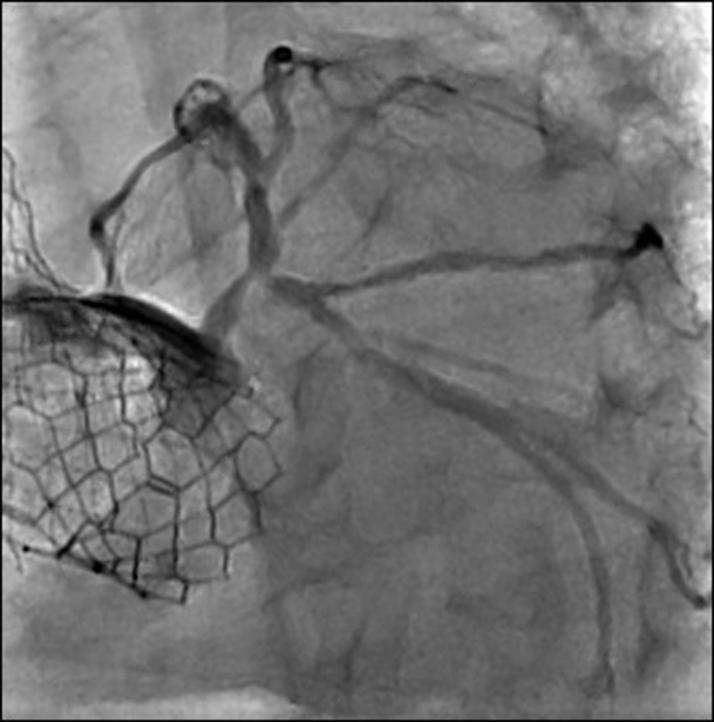
Baseline Coronary Angiography, Nonselective Injection, Spider View Coronary angiography showing a tight stenosis of the distal left main coronary artery involving the ostium of the LAD and the circumflex (Medina 1,1,1). LAD = left anterior descending artery.

**Figure 2:**
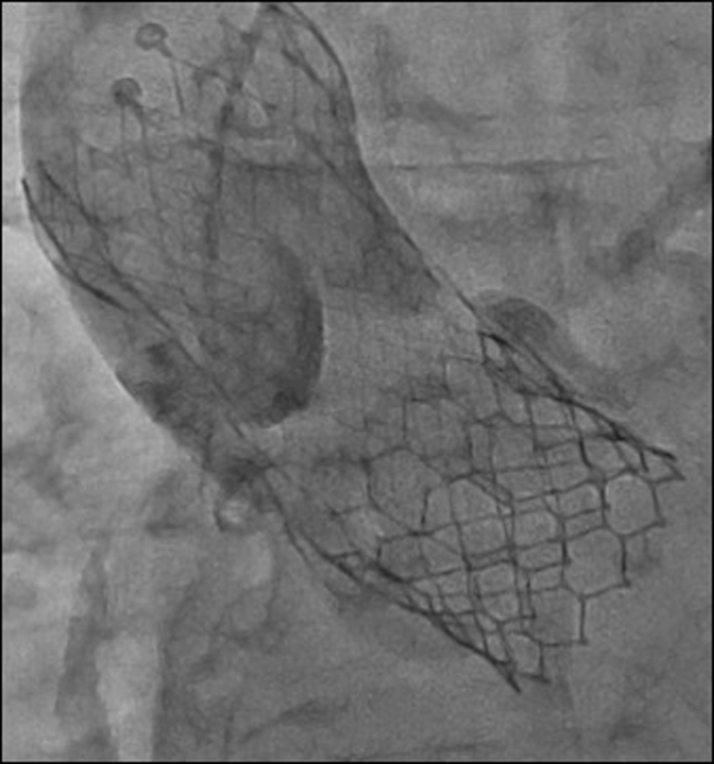
Baseline Coronary Angiography, Nonselective Injection Above the Valve Leaflets RAO caudal projection showing a 5-F pigtail catheter in the ascending aorta, demonstrating that the valve leaflets are above the origins of the coronary arteries. RAO = right anterior oblique.

Right radial coronary angiography was performed, and selective coronary catheterization was not successful. We proceeded through the cells above the coronary ostia using a 0.035-inch guidewire combined with a semiselective injection into the sinus. The right coronary artery was visualized through contrast recirculation between the different aortic sinuses. Despite being nonselective, the opacification allowed us to diagnose triple-vessel coronary artery disease characterized by a severe distal left main artery stenosis and a significant proximal LAD stenosis (Figure 1). Aortic angiography using a pigtail catheter demonstrated that the Evolut valve leaflets were positioned above the origins of the coronary arteries (Figure 2).

**Figure 3:**
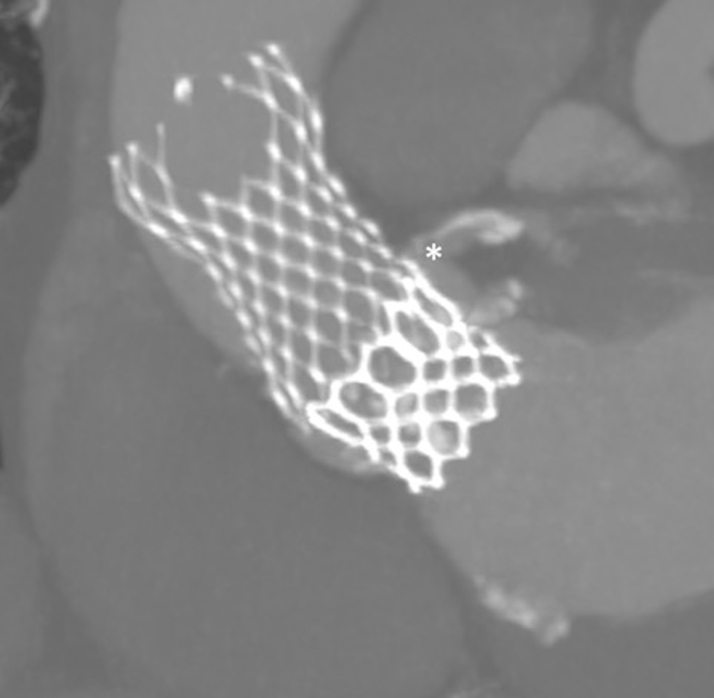
CT Scan, Maximum Intensity Projection The image shows the overlap between the Sapien valve and the Evolut skirt, in front of the left main artery (∗). CT = computed tomography.

**Figure 4:**
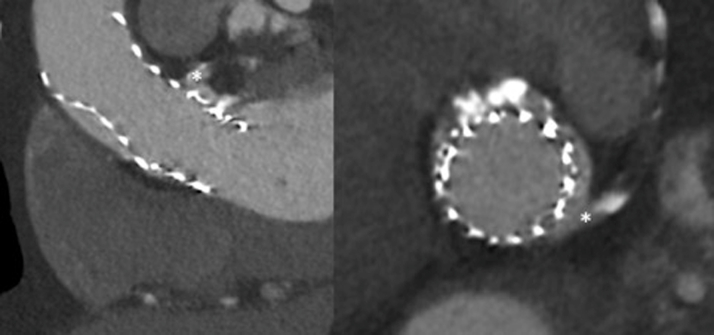
CT Scan, Multiplanar Reconstructions The images show the overlap between the Sapien valve and the Evolut skirt, in front of the left main artery (∗). The valve-to–left main coronary ostium distance measures approximately 3 mm, and the valve-to-sinus junction is virtually absent in the left sinus. CT = computed tomography.

Given the complexity of the coronary lesions and the patient's pain-free status, a cardiac computed tomography scan was obtained (Figures 3 and 4), which revealed an overlap of the 2 valves, with the Sapien valve overlaid by the supra-annular Evolut valve. The overlap of the Evolut's skirt and the Sapien valve resulted in the formation of a neoskirt, which completely obstructed the coronary access at and above the level of the annular plane. Computed tomography further showed a valve-to–left main coronary ostium distance of approximately 3 mm, and a valve-to-sinus junction that was virtually absent in the left sinus. We discussed the case in a heart team meeting. Surgical treatment was formally ruled out, and we decided to propose a percutaneous revascularization to the patient.

Via a left femoral access and using an EBU 3.0 guiding catheter, we navigated beneath the leaflets of the valve, in front of the left main coronary. We connected, using a clamp, the electrosurgical knife (a monopolar generator), to a Steelcore guidewire (Abbott), a hydrophilic polymer-coated coronary guidewire that provides 1:1 torque for precise steering and tip control, insulated in a microcatheter. We then navigated in front of the left main artery, as close as possible to the coronary ostium under fluoroscopic control and under the annular plane of the Evolut valve.

**Figure 5:**
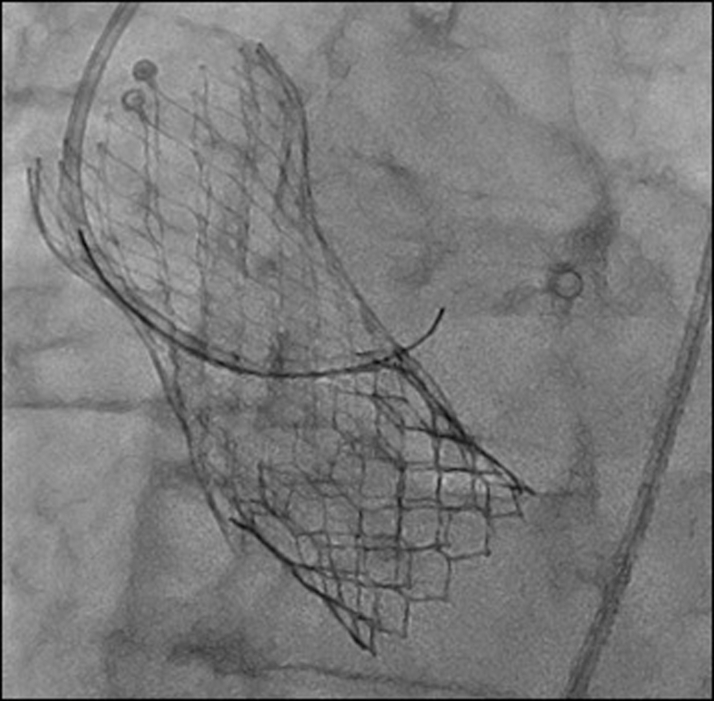
Crossing of the Neoskirt After Electrosurgical Perforation During PCI RAO caudal projection showing the crossing of the skirt by the Steelcore guidewire. PCI = percutaneous coronary intervention; RAO = right anterior oblique.

A slight pressure was applied on the skirt using the Steelcore guidewire and, simultaneously, short electrosurgical bursts at 40 W in cut mode were applied, allowing successful crossing to the left coronary sinus (Figure 5).

**Figure 6:**
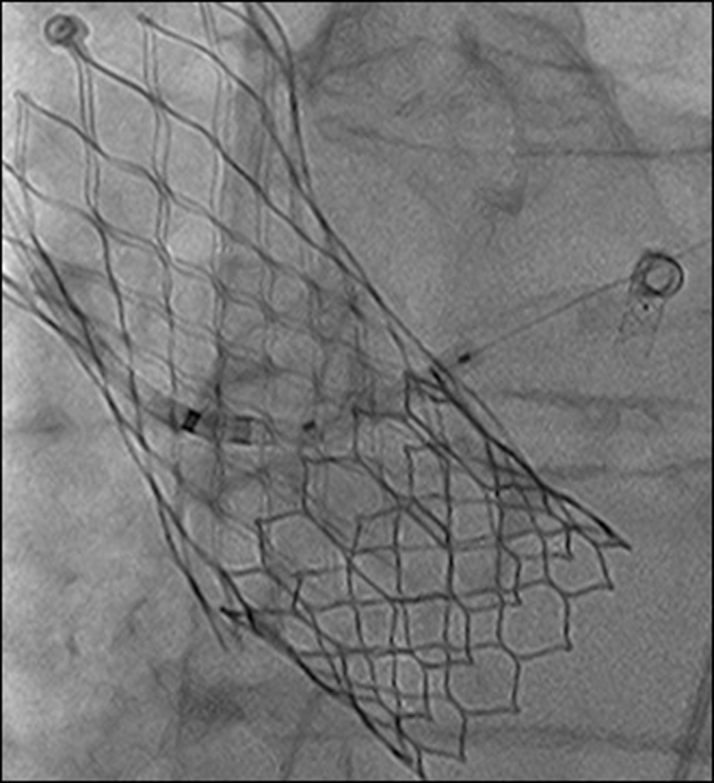
Dilatation of the Neoskirt During PCI RAO caudal showing dilatation of the skirt using a 2.5-mm balloon with subsequent advancement of the guide extension catheter into the ostium of the left main coronary artery. PCI = percutaneous coronary intervention; RAO = right anterior oblique.

**Figure 7:**
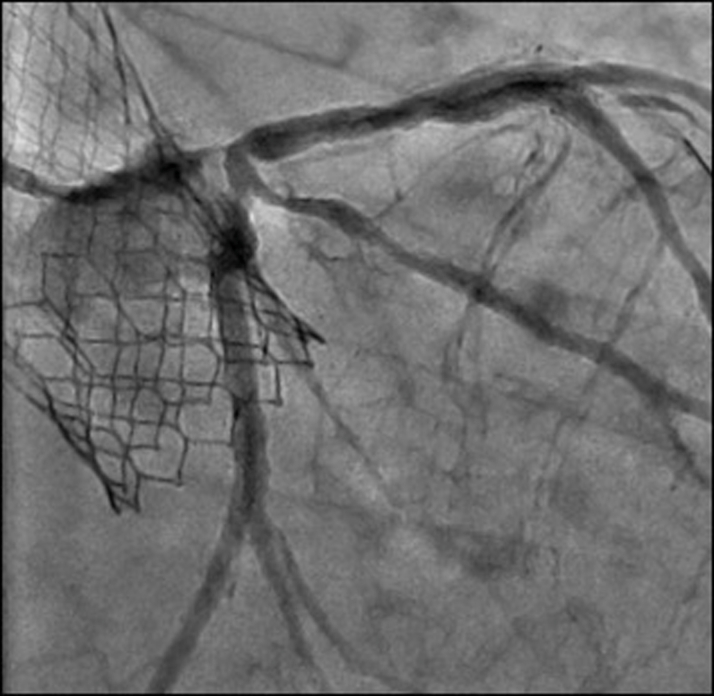
Selective Injection During PCI RAO caudal projection showing selective intubation of the left main artery showing a severe stenosis of the distal left main coronary artery. PCI = percutaneous coronary intervention; RAO = right anterior oblique.

After electrosurgical perforation, a Sion Blue guidewire (Asahi Intecc) was advanced through the stent frame openings into the first diagonal branch. The valve skirt was then dilated using a 1.0-mm followed by a 2.5-mm noncompliant balloon (Figure 6), and a guide extension catheter was advanced through the frame (Figure 7).

A second Sion Blue guidewire was advanced into the circumflex artery. The left main bifurcation was treated using the provisional stenting technique. A 3.5 × 24 mm drug-eluting stent was then implanted from the left main artery into the LAD. The proximal optimization technique was performed using a 4.0-mm balloon. The circumflex artery was rewired, and the stent struts were dilated with a 3.0-mm balloon. Repeat proximal optimization technique of the proximal left main artery was conducted using a 4.5-mm noncompliant balloon.

**Figure 8:**
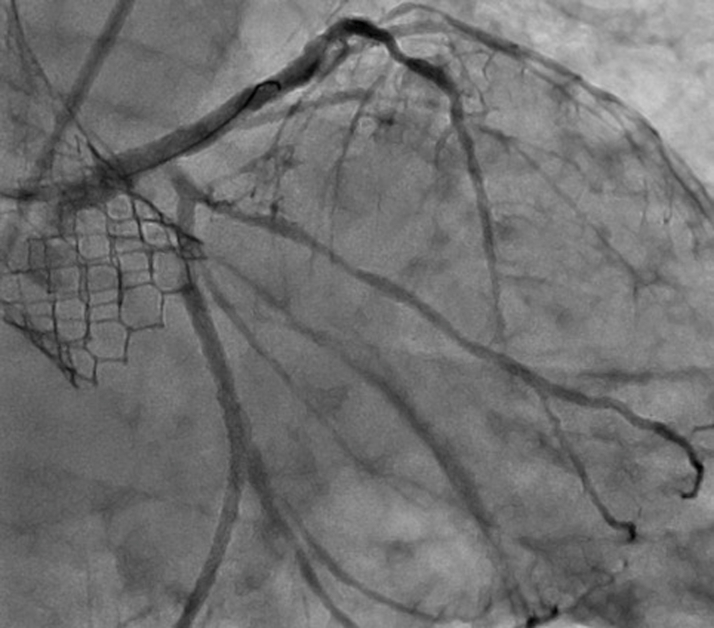
Final Result of PCI Final angiogram after PCI showing a satisfactory result. PCI = percutaneous coronary intervention.

Proximal LAD revascularization was also achieved with the implantation of 2 overlapping drug-eluting stents. The final result was satisfactory (Figure 8).

## Discussion

This case illustrates the feasibility of electrosurgical re-entry through a neoskirt in double TAVI, enabling successful percutaneous coronary intervention when conventional techniques have failed. The rapid rise in the procedural volume of TAVI, coupled with expanding indications to younger and lower risk populations,^5^ implies that a growing proportion of patients will inevitably face progressive coronary artery disease during their lifetime. Additionally, the younger age and prolonged survival of this population increase the likelihood of future reinterventions, including TAVI-in-TAVI procedures, further complicating coronary access.

TAVI-in-valve and TAVI-in-TAVI procedures, which are poised to become routine in clinical practice, further exacerbate the complexity of coronary access. Published data report difficult coronary engagement in approximately 7.7% of all TAVI cases, rising to nearly 18% with supra-annular self-expanding valves.^6^^,^^7^ These anatomical and procedural challenges demand proactive strategies and innovative approaches.

Coronary access after TAVI-in-TAVI may be challenging and should be a point of attention when planning first-time TAVI, especially in younger patients who have a higher likelihood of undergoing TAVI-in-TAVI.^8^ The most concerning point in TAVI-in-TAVI is the creation of a neoskirt by the leaflets of the first valve pushed by the new one, which may lead to coronary sinus obstruction, as illustrated in this case.

While established electrosurgical applications have shown low complication rates when performed with rigorous precautions, extending these principles to neoskirt perforation will require additional experience and follow-up to refine the technique—for example, determining whether skirt dilatation should be performed with a larger balloon—and to fully characterize its safety profile. Reported complications of transcatheter electrosurgery are rare and mainly include perforations or collateral thermal injuries, with their incidence remaining low thanks to rigorous precautions such as continuous fluoroscopic monitoring, precise power settings, and the use of nonionic solutions.^9^

Although still evolving and requiring specific training and careful handling, transcatheter electrosurgery is poised to become an integral component of routine structural heart interventions.

## Conclusions

This case demonstrates the feasibility of transcatheter electrosurgery for coronary revascularization in a challenging access scenario after TAVI. With rising use of TAVI in younger patients with longer life expectancy, the development and standardization of transcatheter electrosurgery will be essential to optimize outcomes.

## Funding Support and Author Disclosures

The authors have reported that they have no relationships relevant to the contents of this paper to disclose.
